# Analysis of Immobilized Protein Unfolding and Molecular Dynamics Shows How pH, Glycosylation, and OCA3-Related Variants Influence Tyrp1’s Stability and Function

**DOI:** 10.3390/ijms27114961

**Published:** 2026-05-30

**Authors:** Waleed Sabir, Isabella Osuna, Monika B. Dolinska, Yuri V. Sergeev

**Affiliations:** Protein Biochemistry and Molecular Modeling Group, Ophthalmic Genetics and Visual Function Branch, National Eye Institute, National Institutes of Health, Bethesda, MD 20892, USA; sabirwaleed01@gwmail.gwu.edu (W.S.); isabellaosuna2@gmail.com (I.O.); dolinskam@nei.nih.gov (M.B.D.)

**Keywords:** Tyrp1, oculocutaneous albinism type 3, protein stability, melanosome, glycosylation, molecular dynamics

## Abstract

Tyrosinase-related protein 1 (Tyrp1) is a melanosomal glycoprotein required for eumelanin biosynthesis through the oxidation of 5,6-dihydroxyindole-2-carboxylic acid (DHICA). Pathogenic variants in Tyrp1 cause oculocutaneous albinism type 3 (OCA3), but the molecular basis by which individual substitutions impair Tyrp1 stability and activity remains incompletely understood. Here, we examined wild-type Tyrp1 and three missense variants associated with OCA3: R356Q and R326H as OCA3-related variants, and D308N as a benign control; these were under conditions relevant to melanosome maturation. To assess stability, we developed a urea-induced unfolding assay in which His-tagged Tyrp1 variants were immobilized to Ni-NTA magnetic beads before chemical denaturation. R356Q was the most destabilized variant, with a ΔΔG of 0.695 kcal/mol at pH 5.0 (acidic conditions) and 1.998 kcal/mol at pH 7.4 (near-neutral conditions) relative to wild-type. R326H showed intermediate destabilization, whereas D308N behaved similarly to wild-type. DHICA oxidation assays in the presence of MBTH showed about 20% reduced catalytic activity for R356Q, particularly under acidic conditions. Molecular dynamics simulations and ligand docking were consistent with these findings and indicated that R356Q increases conformational flexibility and perturbs structural integrity. In contrast, glycosylation reduced conformational fluctuations and enhanced stability across Tyrp1 and mutant variants examined. Together, these results show that pH, glycosylation, and disease-associated substitutions collectively modulate Tyrp1 folding energetics and catalytic competence and identify R356Q as a strongly destabilizing OCA3 variant. By defining how disease-associated Tyrp1 substitutions affect protein stability and function, this study may provide a framework for interpreting genotype–phenotype relationships and improving molecular diagnosis of OCA3.

## 1. Introduction

Melanogenesis within the melanosome produces two major forms of melanin—eumelanin and pheomelanin—which determine the pigmentation of the hair, skin, and eyes. This biosynthesis process involves three key enzymes: tyrosinase (Tyr), tyrosinase-related protein 1 (Tyrp1), and tyrosinase-related protein 2 (Tyrp2). Tyr catalyzes the initial steps of melanin synthesis by converting tyrosine to L-DOPA, which is then converted to dopaquinone, a precursor to dopachrome. Tyrp2 then converts dopachrome to 5,6-dihydroxyindole-2-carboxylic acid (DHICA). Tyrp1 has been proposed to catalyze the oxidation of DHICA to 5,6-indolequinone-2-carboxylic acid (IQCA), although its precise enzymatic function, especially across species, is not well-established [1,2,3]. In murine melanocytes, Tyrp1 functions as a DHICA oxidase, and in contrast, human Tyrp1 does not use DHICA as a substrate for oxidation [3].

Tyrp1 is an essential component in melanin biosynthesis within melanocytes. Along with Tyr and Tyrp2, Tyrp1 contributes to the enzymatic cascade that produces eumelanin (brown/black pigment) and pheomelanin (red/yellow pigment) [2]. Two zinc atoms are in the active site of Tyrp1, which catalyzes a late step in eumelanin synthesis: the oxidation of DHICA to IQCA [4,5]. In addition to its enzymatic role, Tyrp1 is implicated in stabilizing the melanin-synthesizing complex and maintaining melanosome structure. Evidence indicates that Tyrp1 contributes to the maintenance of melanosomal ultrastructure and promotes melanocyte viability [6], implying that its role extends beyond catalysis to include structural or chaperone-like functions.

Human Tyrp1 shares approximately 44% sequence identity with human Tyr [5]. Both are type 1 membrane-bound glycoproteins with the C-terminal alpha helices spanning the membrane of the melanosome. However, their catalytic properties differ: Tyr contains a di-copper active site, whereas Tyrp1 binds two zinc ions in the catalytic cavity instead. Human Tyrp1 is composed of 537 amino acids, including a signal peptide (residues 1–24), localized within the melanosome intra-melanosomal domain (Cys-rich subdomain, residues 25–126), a tyrosinase-like subdomain (residues 127–477), a C-terminal transmembrane-helix (residues 478–501), and a cytoplasmic domain (residues 502–537) [7]. In melanosomes, tyrosinase proteins attach to the membrane via a transmembrane helix; their cytoplasmic domain faces the cytosol, and their catalytic domain is inside the melanosome [8]. Although Tyr, Tyrp1, and Tyrp2 tyrosinases are related proteins and demonstrate a high sequence homology, only the crystal structure of the truncated intra-melanosomal domain of Tyrp1 (residues 1–447) has been resolved [5].

During melanosome maturation, their internal pH gradually rises from an acidic environment in the early stages to more neutral conditions in later stages. In fact, early-stage melanosomes maintain a low pH necessary to regulate the balance between eumelanin and pheomelanin synthesis. In later stages, melanosomes shift to a near-neutral pH, aiding final pigment polymerization and increasing structural stability. Consequently, disruptions in pH balance can cause pigmentation defects. Since Tyrp1 functions within this dynamic pH range, its structure and stability are likely adapted to both acidic and neutral conditions encountered during melanosome maturation. Therefore, studying Tyrp1 under acidic versus near-neutral conditions is important for understanding its true behavior in vivo.

**Figure 1:**
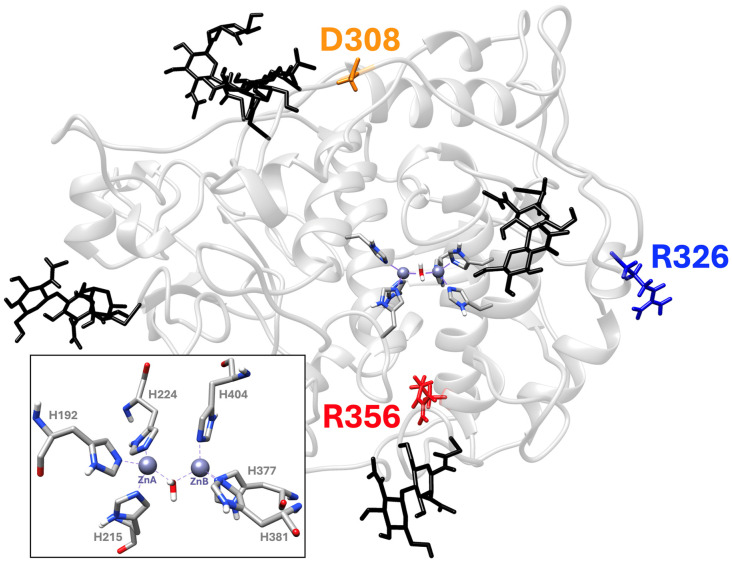
Human recombinant Tyrp1 model obtained using the crystal structure of human Tyrp1 (PDB ID: 5M8L) and visualized by the molecular graphics program UCSF Chimera. The protein structure is shown as a grey ribbon diagram, with three mutation-associated residues (R326, blue; R356, red; D308, orange) represented as stick models. N-linked glycans are shown in black. The inset highlights the active site, including zinc ions (ZnA and ZnB) and the six surrounding histidine residues.

Mutations in Tyrp1 can lead to oculocutaneous albinism type 3 (OCA3), an autosomal recessive disorder characterized by reduced melanin pigmentation. Individuals with OCA3 typically have light brown skin, reddish hair, and ocular pigment alterations, reflecting partial loss of Tyrp1 function [9]. Predominantly affects individuals of African ancestry; it is rare elsewhere. Exact global prevalence is unclear due to underdiagnosis and limited genetic testing. In certain African populations, overall, OCA prevalence (all types combined) can be as high as ~1 in 1000–5000, with OCA3 contributing a notable subset. Often underrecognized due to a milder phenotype and population-specific presentation. To date, the mechanism by which genetic mutations in human Tyrp1 cause OCA3 remains unclear. Known pathogenic Tyrp1 mutations include missense changes that affect protein folding, stability, or catalytic activity. To better understand the mechanism of these genetic perturbations, we examined three Tyrp1 missense variants—R356Q, R326H, and D308N—located in different regions of the protein structure (Figure 1).

In Figure 1, the active site of human Tyrp1, which includes two zinc atoms (ZnA and ZnB) and six coordinating histidine residues, is shown. D308N has been identified in OCA3 patients but retains a near wild-type fold in prior biochemical assays, making it a useful “control” for minimal destabilization of protein structure [7,10]. Although R326H has occasionally been observed in affected individuals, it is classified as benign or likely benign in ClinVar. Computational and limited experimental data predict a modest decrease in stability, greater than the minimal perturbation observed for D308N [7]. In contrast, R356Q has shown conflicting classifications in ClinVar: two classify it as pathogenic, one as likely pathogenic, and another as of uncertain significance, indicating that its impact on protein folding may be more pronounced (https://www.ncbi.nlm.nih.gov/clinvar/variation/17596/, accessed on 10 November 2024).

To assess protein stability, we developed, for the first time, a urea-induced unfolding assay in which His-tagged Tyrp1 variants were immobilized to Ni-NTA magnetic beads before chemical denaturation and successfully measured protein stability in these proteins. To complement the experiments with immobilized proteins, we performed molecular dynamics (MD) simulations and computational analyses. Using the crystal structure of human Tyrp1’s intra-melanosomal domain as the starting model, we simulated the wild-type and mutant variants under neutral and acidic conditions, both in the presence and absence of N-linked glycosylation (https://www.rcsb.org/structure/5M8L/, accessed on 10 November 2024). Tyrp1 is known to be N-glycosylated in vivo, and glycosylation can affect folding stability by stabilizing the protein’s structure [5]. Protonation states of ionizable residues were adjusted in silico to reflect pH conditions. Each system was simulated for 100 ns, and trajectories were analyzed for root-mean-square deviation (RMSD), root-mean-square fluctuations (RMSF) per residue (to identify flexible regions), and conformational free energy landscapes (to visualize stability basins and conformational spread). To further evaluate how structural changes may influence function, we conducted molecular docking experiments using Tyrp1’s substrate, DHICA, and product, IQCA, on representative structures from our simulations. This analysis examined whether mutations or pH changes alter active site geometry or affect ligand binding.

## 2. Results

### 2.1. Experimental Characterization of Human Recombinant Tyrp1 and Mutant Variants Protein Stability

#### 2.1.1. Protein Purification

The human recombinant intra-melanosomal domains were purified twice, once under near-neutral and once under acidic conditions, following the Experimental Procedures. Each cycle involved a two-step purification process. Individual profiles of each protein, Tyrp1, R326H, D308N, and R356Q, were separated into three peaks after the size-exclusion chromatography, as shown in Supplementary Figure S1. Fractions of interest were pooled together for each protein and concentrated with a total protein yield of 6.55, 12.08, 15.92, and 7.08 mg for Tyrp1, R326H, D308N, and R356Q, respectively. The protein identity was confirmed at the proper band length using SDS-PAGE and Western blot analyses.

#### 2.1.2. Catalytic Activity

**Figure 2:**
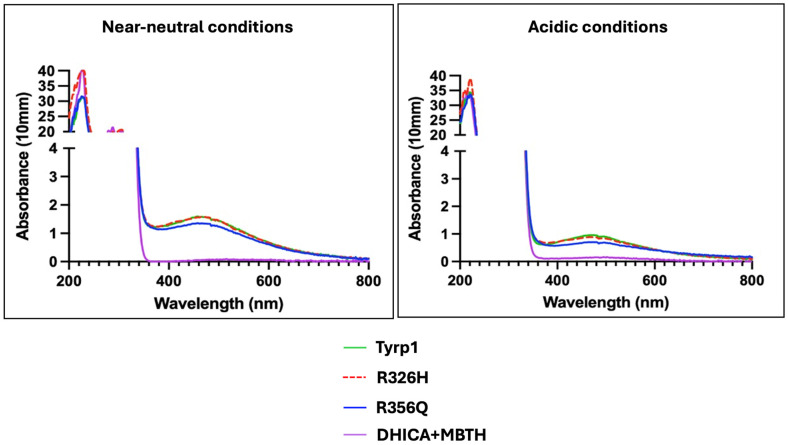
Enzymatic activity of wild-type Tyrp1 and mutant variants (R326H, R356Q) in the presence of MBTH. Experiments were performed in near-neutral (**left**) and acidic (**right**) conditions. The pigment formation was assessed by measuring absorbance at 505 nm using DHICA and MBTH as substrates.

Previously, we tested the catalytic activity of D308N and R326H at neutral pH [10]. Here, we measured Tyrp1 and its mutants (R326H and R356Q) at pH 5.0 and pH 7.0 to compare their enzymatic activity under acidic and near-neutral conditions, respectively (Figure 2). The reaction involving DHICA as a substrate was performed in the presence of MBTH, which forms a chromogenic complex with the oxidized product IQCA. The complex reached peak absorbance at 505 nm, enabling spectrophotometric measurement of enzymatic activity. At near-neutral pH, wild-type Tyrp1 displayed robust enzymatic activity, indicated by a clear absorbance peak around 505 nm, while both mutants exhibited slightly reduced activity. Notably, at acidic pH, enzymatic activity was significantly reduced for all proteins, with the mutants exhibiting minimal activity relative to the wild-type. This reduction may result from impaired zinc coordination and alterations in catalytic geometry, which decrease enzymatic efficiency [11,12]. Among the variants tested, wild-type Tyrp1 remained the most active and stable at both pH conditions. The substrate-only control (DHICA in the presence of MBTH) consistently showed low absorbance, validating the specificity of the enzymatic reactions.

#### 2.1.3. Immobilized Urea-Induced Unfolding Assay

In the Tyrp1 protein structure, nine tryptophan residues could be found, but only three are exposed to the protein surface, and the six residues are buried in the hydrophobic core. Protein unfolding induced by urea exposes these six residues, leading to changes in fluorescence. These changes were monitored spectrophotometrically. Emission during unfolding was recorded from 305–400 nm with an excitation at 285 nm at 0 M and 8 M urea for each protein sample. An example of the emission spectrum from the unfolding experiment is shown in Supplementary Figure S2. A shift in fluorescence from 345 nm to 365 nm was observed when comparing 0 M to 8 M urea for both intact and immobilized protein, confirming the expected tryptophan fluorescence change.

**Figure 3:**
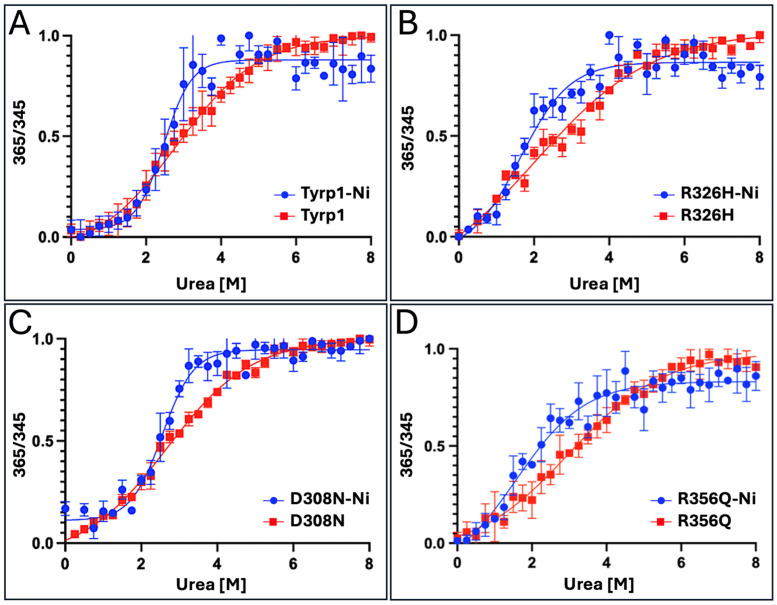
Urea-induced unfolding equilibrium curves and protein stability profiles obtained for immobilized and intact proteins at near-neutral conditions. All Panels show the fluorescence emission ratio (365/345 nm) as a function of urea concentration. Each unfolding curve represents the average of triplicate measurements, with standard errors shown as error bars. Blue solid circles indicate data for proteins immobilized on Ni-NTA particles: Tyrp1-Ni (**A**), R326H-Ni (**B**), D308N-Ni (**C**), and R356Q-Ni (**D**). Red solid squares represent data for the corresponding intact proteins.

The unfolding experiment was repeated under the same conditions using a urea gradient from 0 to 8 M. Urea-induced equilibrium unfolding of Tyrp1 and its mutant variants was obtained by plotting the fluorescence emission ratio (365/345 nm) as a function of urea concentration. The results of the measurement at neutral conditions are shown in Figure 3.

Sigmoidal curves were fitted using Boltzmann functions, which were normalized in a range between 0 and 1. For each mutant variant, R326H, D308N, or R356Q, protein stability, expressed as free energy changes (ΔG°), and the corresponding protein stability changes (ΔΔG) caused by mutations were determined at 0 M urea concentration and are shown in Supplementary Figure S3 and Table 1.

Similar calculations were applied to the corresponding immobilized proteins. In silico stability values were obtained from the reference [7]. A notable difference in stability was observed between intact Tyrp1 (1.979 kcal/mol) and the immobilized form (2.562 kcal/mol), suggesting that immobilization improves estimates of native protein stability. A similar increase was observed for the D308N mutant variant, with stability increasing from 1.863 kcal/mol (intact) to 2.308 kcal/mol (D308N-Ni), giving a ΔΔG of 0.254 kcal/mol (Table 1). For R326H, immobilization results in a slight increase in stability, from 1.560 kcal/mol (intact) to 1.800 kcal/mol (R326H-Ni), with a ΔΔG of 0.762 kcal/mol. Interestingly, the R356Q mutant shows a different pattern. While the stability of intact R356Q is 1.324 kcal/mol, the immobilized form exhibits reduced stability, with a ΔΔG of 1.998 kcal/mol (Table 1), suggesting the unstable mutant variant in immobilized conditions. Urea-induced unfolding experiments are shown in Figure 3.

**Figure 4:**
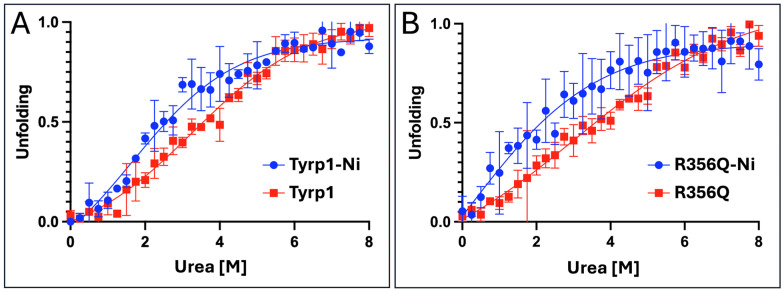
Urea-induced unfolding of wild-type Tyrp1 and R356Q mutant with and without Ni-NTA bead immobilization at acidic conditions. (**A**) Tyrp1 immobilized magnetic beads (blue circles) versus intact protein (red squares). (**B**) R356Q mutant immobilized on magnetic beads (blue circles) versus intact mutant (red squares). Data points represent unfolding fractions from three independent experiments; error bars denote one standard deviation.

Under acidic conditions, wild-type Tyrp1 exhibited greater resistance to unfolding than the R356Q variant, as indicated by a higher midpoint urea concentration in Figure 4A compared with Figure 4B. Immobilization of Tyrp1 on Ni-NTA beads modestly delayed unfolding (blue vs. red curves in Figure 4A), suggesting a stabilizing effect, likely due to restricted conformational mobility upon surface binding. In contrast, R356Q unfolded at lower urea concentrations than the wild-type in both free and immobilized forms (Figure 4B), consistent with reduced stability caused by the mutation. Immobilization provided only partial stabilization of R356Q, shifting the unfolding transition to slightly higher urea concentrations.

Protein stability in water (Supplementary Figure S4) and the corresponding free energy changes (ΔΔG) for intact and Ni-NTA-immobilized proteins are reported in Supplementary Tables S1 and S2, respectively. The intact R356Q variant exhibited stability changes typical of mild mutations, with ΔΔG values of 0.421 kcal/mol and 0.655 kcal/mol under acidic and near-neutral conditions, respectively (Supplementary Table S1). For the Ni-NTA-immobilized R356Q variant (Table 1; Supplementary Table S2), the change in protein stability increased slightly under acidic conditions (ΔΔG = 0.695 kcal/mol) and rose markedly to 1.998 kcal/mol under near-neutral conditions, indicating a stronger destabilizing effect. Overall, urea-induced unfolding experiments demonstrated that the R356Q mutation reduces Tyrp1 protein stability, with a more pronounced destabilizing effect at near-neutral pH.

### 2.2. Computational Analysis of Tyrp1 and Mutant Variants Structures

#### 2.2.1. MD Simulations: RMSD Analysis

**Figure 5:**
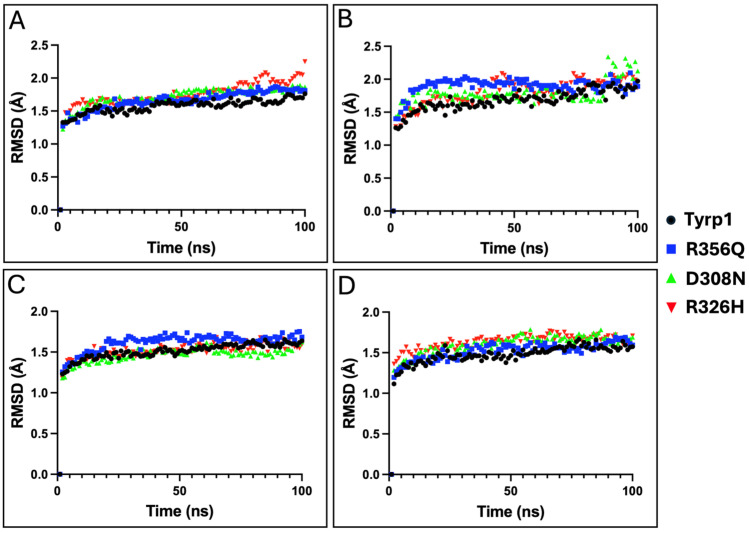
RMSD of wild-type Tyrp1 and associated mutant variants was measured from alpha-carbon atoms over 100 ns MD simulations under different glycosylation and pH settings: (**A**) shows acidic glycosylated, (**B**) acidic deglycosylated, (**C**) near-neutral glycosylated, and (**D**) near-neutral deglycosylated conditions.

MD simulations of Tyrp1 and associated mutant variants were performed under four conditions, and structural stability was assessed using RMSD over 100 ns trajectories (Figure 5).

Under acidic, glycosylated conditions, wild-type Tyrp1 showed the lowest RMSD, indicating the greatest structural stability, with D308N exhibiting a similar behavior. R356Q displayed moderate deviation, whereas R326H underwent the largest structural drift. Upon deglycosylation at acidic pH, RMSD increased in all variants, with R326H and D308N showing the most pronounced destabilization. These findings underscore the stabilizing effect of glycosylation under low-pH conditions.

At near-neutral pH with glycosylation, R356Q exhibited the highest RMSD, indicating substantial instability, whereas wild-type and D308N remained stable, and R326H showed moderate deviation. Under neutral, deglycosylated conditions, all variants displayed the highest RMSDs, demonstrating that the combination of physiological pH and deglycosylation is most destabilizing, with R356Q and R326H showing the largest deviations.

#### 2.2.2. MD Simulations: RMSF Analysis

**Figure 6:**
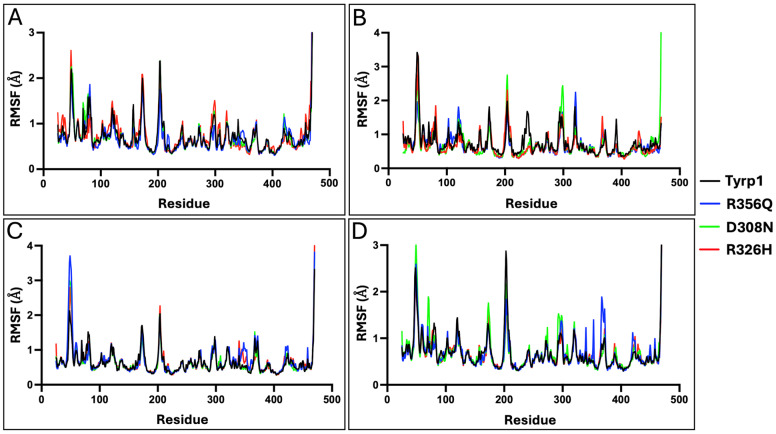
RMSF analysis revealed varying flexibility in Tyrp1 and OCA3 mutant residues based on glycosylation and pH. Regions with higher fluctuations are sensitive to mutation-induced structural changes. (**A**) covers acidic, glycosylated conditions; (**B**) shows acidic, deglycosylated conditions; (**C**) presents near-neutral, glycosylated conditions; and (**D**) illustrates near-neutral, deglycosylated conditions.

Residue-level flexibility was assessed using RMSF (Figure 6). Under glycosylated acidic conditions, wild-type, D308N, and R326H showed low fluctuations, whereas R356Q exhibited increased flexibility, particularly across the fragment of residues 100–200. Deglycosylation at acidic pH increased flexibility in all variants, particularly in loop regions (residues 60–120 and 400–450), with R356Q showing the greatest effect.

At near-neutral pH, all variants show maximum flexibility under neutral, deglycosylated conditions. R356Q causes major instability and greater flexibility; R326H moderately destabilizes; D308N behaves like the wild-type.

#### 2.2.3. MD Simulations: Free Energy Landscapes

**Figure 7:**
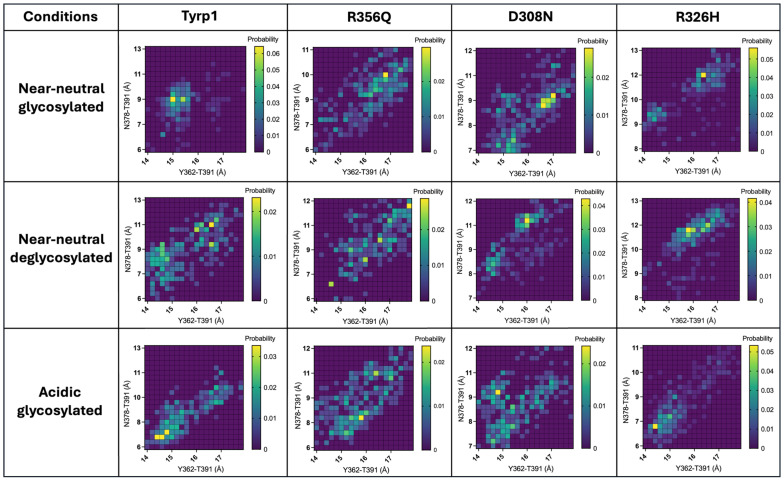
Free energy landscapes for human recombinant Tyrp1 and mutant variants (R356Q, D308N, R326H) are shown under near-neutral glycosylated, deglycosylated, and acidic glycosylated conditions. Axes indicate inter-residue distances Y362-T391 (x) and N378-T391 (y) in Å. The color scale shows conformational probability: yellow marks highly populated states, while darker areas highlight the most stable conformations related to active-site movements.

Two-dimensional free energy landscapes were generated to examine how pH and glycosylation influence the conformational ensembles of Tyrp1 and its mutants (Figure 7).

Wild-type Tyrp1 showed a compact, well-defined energy basin under glycosylated near-neutral conditions, consistent with a stable conformation. Deglycosylation resulted in a broader basin, signifying diminished structural stability. Conversely, glycosylated proteins at acidic pH exhibited a subtle shift and increased expansion, which implied modified yet partially preserved stability. R356Q exhibited the most pronounced disruption, with a broad, shifted energy landscape spanning a wide range of conformations, indicative of high flexibility and loss of structural stability. In contrast, D308N closely resembled the wild-type, maintained a narrow, well-defined basin, and near-native conformational stability. R326H displayed intermediate behavior, with a moderately broadened and slightly shifted basin, reflecting partial destabilization while still favoring a compact state.

Deglycosylation of wild-type Tyrp1 broadened the low-energy region, indicating structural destabilization and reduced preference for a single compact state. Simulating the glycosylated wild-type at acidic pH produced a wider, shifted energy basin compared to near-neutral pH, but it remained compact; this shift revealed that pH influences conformational preference.

Among the mutants, R356Q showed the most dramatic alteration in the free energy landscape, matching our results from urea-induced unfolding experiments. The R356Q mutant’s low-energy region was extremely broad and shifted relative to the wild-type basin, covering a larger range of inter-residue distances. This indicated that the mutant adopted multiple distinct conformations with similar probabilities, reflecting a highly flexible and unstable structural ensemble. R356Q substitution broke native contacts, causing loss of the wild-type’s stable conformation. R356Q failed to attain a compact energy minimum, including in its glycosylated form; moreover, the absence of glycan or exposure to acidic pH conditions was anticipated to further enhance its molecular flexibility. The free energy landscape of the D308N mutant closely resembled that of wild-type Tyrp1. The low-energy basin for D308N remained narrow, compact, and centered in nearly the same position as the wild-type basin under equivalent conditions. This indicated that the D308N substitution did not significantly perturb the protein’s conformational preferences or stability. Glycosylated D308N at near-neutral pH retained a well-defined energy minimum, implying that the mutant protein maintained a stable conformation closely resembling the native state.

The R326H mutant showed intermediate behavior, between the stability of D308N and the instability of R356Q. While the free energy landscape preserved the main low-energy basin, it showed some broadening and a small shift compared to the wild-type. The conformational basin became more elongated, indicating a wider range of sampled distances, while favoring a compact state. This suggests R326H disrupted stabilizing interactions without fully destabilizing the structure. Under glycosylated, near-neutral pH conditions, R326H maintained a discernible, though less well-defined, energy minimum. Deglycosylation or further lowering pH disrupted the landscape, but not as much as R356Q.

#### 2.2.4. Molecular Docking

Molecular docking of the Tyrp1 substrate DHICA was performed to assess how the observed structural differences translate into functional outcomes. Docking of wild-type and mutant Tyrp1 under various conditions estimated binding energies and revealed how mutations and environmental factors affected substrate positioning in the active site. Docking simulations revealed notable differences in DHICA binding across glycosylation states and pH conditions (Supplementary Figure S5). Deglycosylation at near-neutral pH resulted in decreased binding affinity, indicating that glycosylation played a key role in maintaining active-site conformation and promoting optimal ligand alignment. Under acidic conditions with glycosylation, binding energies were intermediate, suggesting partial disruption of stabilizing interactions.

Among the mutants, R356Q exhibited consistently higher binding energies across all conditions, particularly under glycosylated, neutral conditions, indicating impaired ligand binding. Structural analysis suggested that this mutation increased conformational flexibility and enlarged the active site, allowing greater spatial freedom for DHICA while reducing binding precision and affinity. This observation was consistent with the free energy landscape analysis, in which R356Q displayed a broad basin spanning a wide range of inter-residue distances. In contrast, D308N showed binding energies comparable to those of the wild-type under all conditions. R326H exhibited intermediate effects, with reduced binding affinity relative to the wild-type, most notably under deglycosylated, near-neutral conditions.

## 3. Discussion

This study examines how the melanosomal environment and OCA3-associated mutations shape the stability and function of Tyrp1. By analyzing the protein under acidic and near-neutral pH conditions, comparing glycosylated and non-glycosylated forms, and evaluating wild-type Tyrp1 alongside the R356Q, D308N, and R326H variants, this work identifies key factors that govern protein behavior and dysfunction. Together, the results suggest that both extrinsic conditions, such as pH, and intrinsic factors, including post-translational modification and amino acid substitution, contribute to Tyrp1 destabilization and loss of activity. Framing these findings in terms of pH-dependent effects, the protective role of glycosylation, and the distinct structural consequences of each mutation provides a mechanistic basis for understanding how these variants may drive OCA3 pathogenesis. In this work, most findings come from molecular simulations, chemical denaturation, immobilized proteins, and recombinant Tyrp1 constructs, not native melanocytes or in vivo models. Thus, the results may not reflect the full complexity of physiological melanosomes.

### 3.1. Tyrp1 Catalytic Activity

The catalytic function of Tyrp1 remains a subject of ongoing debate [3,5]. ICP-MS spectroscopy showed that human recombinant Tyrp1 and its benign mutants (D308N, R326H) have significant active site zinc occupancy, with Zn^2+^ roughly 75 times higher than Cu^2+^. In contrast, the more severe OCA3-associated variants (H215Y, R87G, and C30R) showed a marked reduction in zinc content, which correlated with diminished enzymatic activity. These findings suggest that human recombinant Tyrp1 is catalytically active when Zn^2+^ ions occupy the active site. DHICA oxidase activity of human Tyrp1 may be influenced by assay conditions, MBTH cross-reactivity with melanogenesis intermediates, or temperature. In the present study, DHICA oxidase activity was observed in human Tyrp1 and mutant variants at 37 °C. In contrast, in previous a study [5] measurements reporting no DHICA oxidase activity in zinc-human Tyrp1 were performed at 298 K (25 °C). Although MBTH is selective for quinones such as IQCA, its broader reactivity with other melanogenesis intermediates, such as indole-5,6-quinone and dopaquinone, may limit specificity and should be considered in future analyses [13].

### 3.2. Immobilized Tyrp1

In our previous study, Tyr was immobilized onto Ni-NTA particles through a 6 × His tag [11]. Transmission electron microscopy (TEM) revealed that the Tyr-immobilized particles had an average diameter of 168.2 ± 24.4 nm. By contrast, dark-brown melanin structures were observed in both monomeric and polymerized forms, with an average diameter of 121.4 ± 18.1 nm. Hill kinetic analysis demonstrated that immobilized Tyr retained residual catalytic activity at 50 °C, indicating enhanced thermal stability. These results indicate that immobilized human Tyr remains catalytically active at the nanoscale and exhibits greater thermal stability than the non-immobilized enzyme.

### 3.3. Protein Stability in Melanosomal Environments

Melanosome maturation is accompanied by a shift in luminal pH from acidic in early-stage melanosomes to near-neutral in mature melanosomes [14]. This transition has important implications for Tyrp1 structure and function. Under acidic conditions, key histidine residues in the active site become protonated, weakening their coordination to the binuclear Zn^2+^ cofactors. Tyrp1 contains six histidines that coordinate two Zn^2+^ ions, and protonation of these ligands is expected to disrupt metal positioning and active-site geometry [5]. As a result, enzymatic activity is reduced in early melanosomes, likely serving as a regulatory mechanism to suppress DHICA oxidase activity until the pH increases [15]. Consistent with this model, our simulations at acidic conditions revealed subtle destabilization even in wild-type Tyrp1, including modest increases in RMSD and localized flexibility. The results indicate that the protein undergoes conformational strain in acidic conditions, consistent with disrupted active-site coordination. The R356Q mutant showed heightened sensitivity to acidic conditions, with increased RMSF and reduced structural rigidity. Wild-type and milder variants show low fluctuations, indicating partial tolerance to acidic stress due to glycosylation and disulfide bonds. Destabilized variants like R356Q lack this resilience.

As the melanosomal pH approaches neutrality, Tyrp1 adopts a more stable and catalytically competent conformation. Wild-type Tyrp1 remains stable at neutral pH, but disease-linked mutants do not regain full stability. Chemical denaturation experiments revealed that both wild-type Tyrp1 and the R356Q variant exhibit increased overall stability under near-neutral conditions. The acidic environment (~0.7 kcal/mol) partially masks the mutation’s effect, whereas near-neutral pH exposes its full destabilizing impact (~2 kcal/mol). This trend is supported by MD simulations, in which wild-type Tyrp1 maintained a stable fold at near-neutral pH, while R356Q exhibited substantial conformational drift. Collectively, these findings indicate that acidic conditions impose baseline structural stress on Tyrp1, particularly in destabilized variants, while near-neutral pH is required for full structural stabilization, proper Zn^2+^ coordination, and enzymatic activity.

Evaluating changes in protein stability within the melanosomal environment is challenging. However, both the intra-melanosomal domain attached to Ni-NTA particles and the domain linked to the transmembrane helix embedded in the melanosome membrane are expected to have similarly restricted mobility, as shown in Supplementary Figure S6. Therefore, experiments with Ni-NTA particles suggest that stability estimates for immobilized tyrosinase are likely to approximate those of the intra-melanosomal domains within the native melanosome environment. Although immobilization on Ni-NTA particles approximates restricted mobility, the model does not fully reproduce melanosomal membrane composition. Such important properties as crowding effects, ion concentrations, redox conditions, or interactions with other melanogenic proteins are excluded from consideration.

### 3.4. Effect of Deglycosylation on Tyrp1 Stability

Tyrp1 is a heavily glycosylated melanosomal enzyme whose native folding and stability depend strongly on N-linked glycans [16,17,18,19,20]. N-linked glycosylation is essential for the proper folding and stability of Tyrp1, which contains six glycosylation sites within its luminal domain [18]. In Tyrp1 mutant variants, glycosylation likely acts as a compensatory structural mechanism that partially offsets destabilizing effects introduced by pathogenic amino acid substitutions. For the D308N variant, glycosylation may preserve hydration networks surrounding nearby loops and secondary structural elements. For the R326H variant, the perturbation reduces the strength and geometry of long-range ionic interactions because histidine is only partially protonated at physiological pH. The glycans, therefore, preserve tertiary structure despite disruption of local electrostatic interactions. For the R356Q variant, the substitution removes a strong electrostatic interaction. Glycosylation may be compensated for by shielding exposed hydrophobic regions, stabilizing partially unfolded conformations, and reducing susceptibility to ER-associated degradation. Across all simulations, glycosylated Tyrp1 exhibited lower RMSD values and a more confined free energy minimum, whereas deglycosylated forms showed greater structural deviation and broader energy landscapes, indicating reduced stability. Deglycosylation increased conformational flexibility across all variants and conditions, promoting sampling of partially unfolded or expanded states. This effect was particularly pronounced at near-neutral pH, where the protein is otherwise more stable and therefore more sensitive to destabilizing perturbations. For example, deglycosylated Tyrp1 at near-neutral pH showed broad free energy distributions, indicating reduced conformational constraint. In contrast, glycosylated Tyrp1 remained confined to a well-defined native basin. These findings have important pathological implications. Disruption of glycosylation, whether due to mutation or cellular stress, can impair proper folding and promote retention of Tyrp1 in the endoplasmic reticulum, where misfolded glycoproteins are often targeted for deglycosylation and subsequent degradation [21]. Our data indicate that deglycosylated Tyrp1 is highly unstable and rapidly loses structural integrity. In contrast, proper glycosylation substantially enhances stability, even in mildly destabilizing mutants.

### 3.5. Mutant Variants: R356Q, R326H, and D308N

The D308N variant showed RMSD and RMSF profiles like those of the wild-type under all tested conditions, with substantial overlaps in the free energy landscape, indicating no significant change in conformational sampling or overall structural dynamics. In contrast, R326H exhibited a modest increase in flexibility and a slightly broader conformational ensemble under near-neutral conditions, consistent with mild destabilization. Despite this effect, R326H caused only a slight reduction in substrate-binding affinity, suggesting minimal perturbation of the active-site environment. These findings are consistent with the benign or likely benign classification of R326H and with our previous reports [7,10].

In contrast to D308N and R326H, the R356Q variant had a pronounced destabilizing effect on Tyrp1, indicating that arginine 356 is important for maintaining structural integrity. Experimentally, R356Q showed reduced folding stability (ΔG°), particularly at near-neutral pH, while computational analyses revealed increased global deviation, enhanced local flexibility, and a broadened free energy landscape, all consistent with a less stable conformational ensemble. A likely mechanism is disruption of the local electrostatic network around residue 356. In the wild-type protein, R356 forms stabilizing interactions with D343 and E360 within the S354-G361 segment. Substitution with glutamine removes the positive charge at this position and abolishes key contacts, including D343(OD2)-R356(HH2) and R356(HH1)-E360(OD1), thereby weakening local structural stabilization. During 10 ns molecular dynamics simulations, this effect resulted in each subunit losing over 80 hydrogen bonds, which suggests a significant breakdown in the intramolecular interaction network. These perturbations likely affect the α-helix spanning residues 375–385, which include H377 and H381, both coordinating the catalytic ZnB ion. Because this region is highly conserved, its destabilization may impair active-site organization, promote misfolding, and ultimately contribute to defective melanogenesis. In this work, all molecular dynamics simulations were done with a limited simulation timescale. The MD simulations are relatively short for capturing large-scale conformational rearrangements, long-timescale unfolding events, or rare transitions. Longer simulations could reveal additional structural dynamics of mutant variants.

### 3.6. Protein Stability and Clinical Significance of Tyrp1 Pathogenic Variants

The severity of the mutant variants was evaluated using protein stability measurements, global computational mutagenesis performed on atomic-level protein structures [7,22,23,24], and clinical significance data (Table 2). Analysis of the Tyrp1 crystal structure suggests that the D308N and R326H variants have little effect on protein stability when compared to the wild-type enzyme. For R326H, this prediction is consistent with its benign clinical classification.

The urea unfolding experiment and predicted foldability parameter from Table 2 both indicate that the R356Q mutant variant is unstable and likely pathogenic. Indeed, the Tyrp1 variant NM_000550.3: c.1067G>A (p. Arg356Gln; R356Q) is reported in ClinVar with conflicting interpretations; however, several recent submissions classify it as pathogenic. This missense substitution has been identified in individuals with OCA3 in both case reports and the scientific literature. Functional evidence, including reduced catalytic activity, altered protein unfolding energetics, and molecular modeling, indicates that this variant disrupts Tyrp1 function, supporting its pathogenicity. Population data further support this conclusion, as the variant occurs at a low allele frequency (~0.01% in gnomAD), consistent with a rare autosomal recessive disorder. Deleterious missense and loss-of-function variants in Tyrp1, such as R356Q, have been linked to OCA3, a condition distinguished by reduced pigmentation of the skin, hair, and eyes. The destabilizing effect of the R356Q mutation is more pronounced in immobilization-based experiments than in assays using intact protein (Supplementary Table S1). Reduced conformational freedom upon immobilization may enhance active-site accessibility and increase apparent reaction rates [23].

The applicability of this approach is constrained by the quality of the available atomic protein structures, the accessibility of reliable phenotypic data, and the inherent limitations of in vitro experimental systems. Nevertheless, our results demonstrate strong concordance among in vitro measurements of protein stability, reverse phenotyping predictions derived from protein atomic structure, and clinical variant classification. Collectively, these findings support the utility of computational mutagenesis as a rapid and effective approach for predicting mutation severity and facilitating computational drug design. Furthermore, the observed stability of immobilized Tyrp1 variants correlates with the clinical significance of pathogenic mutations, providing a mechanistic link between atomic-level perturbations in protein structure and disease phenotype.

## 4. Materials and Methods

### 4.1. Protein Purification

Protein purification and characterization of Tyrp1 and its mutant variants (D308N, R326H, and R356Q) were performed as previously described [7]. The recombinant human intra-melanosomal domains were expressed using a baculovirus system in *Trichoplusia ni* larvae (Allotropic Tech, LLC, Halethorpe, MD, USA). Two separate cycles of complete protein purification were performed under near-neutral and acidic conditions. These cycles were labeled pH 7.4 (near-neutral condition) and pH 5.0 (acidic condition), respectively, indicating that all steps in the first purification were conducted at pH 7.4, while the second cycle was carried out entirely at pH 5.0. For buffers used in both workflows, the pH conditions are indicated as pH 7.4/pH 5.0, corresponding to the values used in the first and second purification cycles, respectively.

The human recombinant intra-melanosomal domains were expressed and produced using the baculovirus in *T. ni* larvae (Allotropic Tech, LLC, Halethorpe, MD, USA). The biomass was homogenized in lysate buffer (20 mM NaPO_4_, pH 7.4/pH 5.0, 500 mM NaCl, 5 mM imidazole, 25 μM 1-Phenyl-2-thiourea, PTU (Sigma-Aldrich, Saint Louis, MO, USA), 2 mM MgCl_2_, 40 μg/mL DNAse I (Thermo Fischer Scientific, Waltham, MA, USA), 0.2 mg/mL lysozyme, and protease inhibitors (Roche, San Francisco, CA, USA)), and then incubated for 30 min at 25 °C, sonicated for 10 min, and centrifuged at 8000 RPM at 4 °C. The final lysate was then diluted with affinity binding buffer (20 mM NaPO_4_, pH 7.4/pH 5.0, 500 mM NaCl, 20 mM imidazole). The lysate was then purified in two steps using an ÄKTAxpress liquid chromatography system and analyzed using the UNICORN 5.31 software (GE Healthcare, Silver Spring, MD, USA). In the first step, the sample was purified using an immobilized metal affinity column (IMAC), His-Trap 5 mL Crude Column (GE Healthcare, NJ, USA), and eluted with affinity elution buffer (20 mM NaPO_4_, pH 7.4/pH 5.0, 500 mM NaCl, 500 mM imidazole) and consecutively applied by the system onto the second step size-exclusion chromatography (SEC) column. The SEC used a HiPrep 26/60 Sephacryl S-300 column with gel filtration buffer (50 mM Tris-HCl, pH 7.4/pH 5.0, 1 mM ethylenediaminetetraacetic acid (EDTA), 50 μM TCEP, 150 mM NaCl). Elution fractions were assessed by SDS-PAGE and Western blot using anti-Tyrp1 antibody TA99 at 1:500. Peak fractions were pooled, concentrated, and stored at 4 °C for short-term use or at −80 °C for long-term storage.

### 4.2. Enzymatic Assay

Two enzymatic assays were performed. In the first one, Tyrp1 and its mutant variants were used at a concentration of 1.0 mg/mL and incubated with 1.0 mM DHICA (Toronto Research Chemicals, Toronto, ON, Canada) and a pH 7.4/pH 5.0 solution of 1.5 mM 3-methyl-2-benzothiazolinone hydrazone hydrochloride (MBTH). Before conducting the assay, protein concentrations were standardized by assessing absorbance at 280 nm and adjusting each sample to an optical density of 1.0. The reaction between MBTH and the oxidation product IQCA resulted in the formation of a dye complex over 4 h, which was monitored by measuring absorbance at 505 nm. Absorbance spectra were recorded over a range of 200–900 nm using a NanoPhotometer N60-Touch (Implen, Westlake Village, CA, USA). Enzymatic reactions were conducted at 37 °C while shaking at 270 rpm. All experiments were performed in triplicate, and the reported values represent the average results.

### 4.3. Protein Immobilization to Ni-NTA Particles

After two cycles of purification, Tyrp1 and its mutant variants were immobilized on Ni–NTA (His-tag affinity) magnetic beads. The beads (Lot: ABCL042723BL) were ordered and customized from Advanced Biochemicals (Lawrenceville, GA, USA). Tyrp1, D308N, R326H, and R356Q (3.0 mg/mL) were immobilized to the beads with binding buffer (1 mM Imidazole, 0.5 M NaCl, 20 mM Tris-HCl, pH 7.4). Ni-NTA beads stock concentration 50 mg/mL) were used at 100 µL of bead slurry (5 mg of beads) per reaction. Each reaction received 200 µL of recombinant protein at 3.0 mg/mL (0.6 mg total), corresponding to a loading of approximately 0.12 mg of His-tagged protein per mg of beads (equivalent to 6 mg protein per mL of bead slurry). The samples were incubated at room temperature for 30 min and rotated on a Roto-Mini Rotator (Benchmark Scientific, Sayreville, NJ, USA). The supernatant was removed using a magnetic separator (Qiagen, Germantown, MD, USA), and the beads with immobilized proteins were treated 3 times with wash buffer (5 mM imidazole, 0.25 M NaCl, 0.05% Tween-20, 10 mM Tris-HCl, pH 7.4) for 5 min at room temperature while rotating. The supernatant was removed, and the wash step was repeated twice. Finally, the magnetic beads were resuspended in 10 mM NaPO_4_ (pH 7.4) before proceeding to the unfolding assay.

### 4.4. Urea-Induced Protein Unfolding and Protein Stability

After the resuspension, the Ni-NTA-immobilized and intact proteins (2.5 µM) were incubated for 24 h at room temperature in 0–8 M urea with a gradient step of 0.5 M. The Ni-NTA protein samples were rotating during the 24 h incubation with urea to fully suspend the beads in the solution, allowing urea to interact with the protein bound to the beads. The samples were plated in a 384-well black, flat-bottom plate (Greiner Bio-One, Monroe, NC, USA) and shaken linearly for 10 s at medium intensity. Protein unfolding was monitored by measuring fluorescence emission from 305–400 nm upon excitation at 285 nm using a SpectraMax i3 (Molecular Devices, CA, USA). Proteins immobilized to Ni-NTA particles were taken from three different immobilizations before incubation with urea. All fluorescence spectra were corrected for the buffer baseline.

### 4.5. Statistical Procedure

Each unfolding curve represented by the ratio 365/345 nm was averaged in triplicate, normalized to show a change in unfolding fraction (u) from 0 to 1, plotted against the concentration of urea, and fit using a Boltzmann function in GraphPad Prism software (version 10.2.2) in triplicate. For each measurement of triplicate, the dependence of the unfolding fraction u on the urea concentration was converted to a free energy plot using the following formula: ΔG = RT ln (u/(1 − u)). Triplicate data points from the free energy plots were averaged, and standard deviations were calculated. Protein stability in water for both intact and immobilized samples was determined as the Gibbs free energy change at zero urea concentration. These values were obtained by linear extrapolation of the experimental data using GraphPad Prism.

### 4.6. Molecular Dynamics Simulations

All-atom molecular dynamics simulations were conducted using the crystal structure of human Tyrp1 (PDB ID: 5M8L), comprising residues 19–476 of the intra-melanosomal domain. Variants modeled included wild-type Tyrp1 and the R356Q, R326H, and D308N mutants, each prepared in glycosylated and deglycosylated forms. A computer program for molecular visualization, modeling, and dynamics, YASARA Structure (https://www.yasara.org, accessed on 10 January 2025), was used [28,29] with the AMBER14 force field incorporating Zn^2+^ parameters specific to the active site. Each structure underwent initial minimization and equilibration phases. Production simulations were conducted at 310 K for 100 ns employing a Langevin thermostat and Berendsen barostat. Simulations for wild-type and the R356Q mutant under glycosylated acidic conditions were performed in triplicate, with distinct random seeds incremented by ±1 in the YASARA macro (md_run). Single replicates were run for all other conditions. Coordinates were recorded every 100 ps throughout simulations. Additional simulations were executed at physiological neutral and acidic pH conditions to assess environmental effects.

### 4.7. Free Energy Landscapes

Free energy landscapes were generated for Tyrp1, D308N, and R326H to assess the conformational stability of the active-site bottleneck. The bottleneck residues were identified as Y362, N378, and T391, as previously done when studying Tyrp1 [7]. The free energy surface was calculated using the two-dimensional weighted histogram analysis method (WHAM), with the Y362(O)–T391(OG1) and N378(OD1)–T391(OG1) distances used as reaction coordinates.

Trajectory analyses were performed using the visual molecular dynamics program VMD 1.9.3 [30] and custom Python 2.7 scripts. The label function in VMD measured the chosen distances at each simulation snapshot for every trajectory. Each system was simulated in triplicate, generating three independent datasets. Distance values were binned into 21 intervals spanning from 4 Å to 14 Å. The WHAM 2D-analysis was performed with a tolerance of 10^−5^, a temperature of 298 K, no padding values (0) to reflect non-periodic reaction coordinates, and a spring constant of 0 (http://membrane.urmc.rochester.edu/?page_id=126, accessed on 10 January 2025). The maximum value and binning parameters were set based on the specific characteristics of each dataset. Free energy was calculated from the probability distributions using the Boltzmann relation to identify the most probable conformational states of the active site.

RMSD and RMSF were computed for Cα atoms to assess global and local dynamics (https://www.ks.uiuc.edu/Research/vmd/, accessed on 10 January 2025).

### 4.8. Ligand Docking Simulations

Ligand docking simulations were performed using YASARA Structure with a custom-modified macro based on the AutoDockLS protocol (https://www.yasara.org, accessed on 10 January 2025). The number of docking runs increased to 250 to enable broader conformational sampling. Receptor and ligand structures were loaded as separate files into a simulation cell centered around the ligand’s alcohol group, positioned between two catalytic zinc ions, with a 10 Å buffer cell. The AMBER14 force field was used to assign partial charges and define ligand flexibility, while receptor side chains were kept rigid. Docking poses were clustered using a 5.0 Å RMSD cutoff. Only poses falling within 4 Å of the active site and properly oriented toward the binuclear zinc center were retained for binding energy analysis. The AutoDockLS scoring function was used within YASARA Structure, with the AMBER14 force field assigning partial charges and defining ligand flexibility; receptor side chains were kept rigid. AutoDockLS was selected over standard AutoDock because its local-search refinement enables broader and more thorough conformational sampling around an initial pose, which was appropriate given the active-site flexibility observed across pH and glycosylation conditions in our MD trajectories. The number of runs increased to 250 to ensure convergence of binding energy estimates. Docking was performed across three structural snapshots (30, 35, and 40 ns) representing pH 7.0, no glycan, pH 7.0 with glycan, and pH 5.0 with glycan. These points were selected based on RMSD stability and equilibration. Final binding energies represent the average of accepted poses across all three frames.

## 5. Conclusions

Using an immobilized unfolding assay together with enzymatic measurements and molecular dynamics simulations, we showed that pathogenic Tyrp1 variants differ in their effects on stability and activity, with R356Q producing the strongest defect. These findings identify melanosomal pH and glycosylation as key determinants of Tyrp1 behavior and provide a biochemical framework for interpreting the clinical significance of Tyrp1 variants.

## Figures and Tables

**Table 1:** Free energy changes for intact and immobilized proteins in neutral conditions.

	**Intact molecules**			
	**Tyrp1**	**D308N**	**R326H**	**R356Q**
ΔG°, kcal/mol	1.979	1.863	1.560	1.324
ΔΔG, kcal/mol	n/a	0.116	0.419	0.655
	**Molecules immobilized to Ni-NTA particles**			
	**Tyrp1-Ni**	**D308N-Ni**	**R326H-Ni**	**R356Q-Ni**
ΔG°, kcal/mol	2.562	2.308	1.800	0.564
ΔΔG, kcal/mol	n/a	0.254	0.762	1.998

**Table 2:** The effect of mutations is evaluated from the variant protein stability, computational mutagenesis, and the clinical significance of the mutation.

Mutation	Mutant Variant of Tyrp1 Intra-Melanosomal Domain		Computational Mutagenesis		ClinVar **	GnomAD **
	ΔΔG, kcal/mol	Fraction of Unfolded Protein	Foldability	Predicted Phenotype		
D308N *	0.254	0.29	0.98	Benign	n/a	n/a
R326H *	0.762	0.87	6.74	Benign	Likely Benign	Benign/Likely Benign
R356Q	1.998	1.00	19.00	Pathogenic	Pathogenic/Likely Pathogenic	Likely Pathogenic

In computational mutagenesis [25,26,27] the mutation is pathogenic if Foldability = 19.9. In silico values were obtained from the reference [7]. * Confirmed in vitro [7,10]. ** ClinVar, ClinVar (nih.gov); gnomAD, gnomAD (broadinstitute.org).

## Data Availability

The original contributions presented in this study are included in the article/Supplementary Materials. Further inquiries can be directed to the corresponding author.

## Supplementary Materials

The following supporting information can be downloaded at: https://www.mdpi.com/article/10.3390/ijms27114961/s1.

## Abbreviations

Tyrp1	tyrosinase-related protein 1
OCA3	oculocutaneous albinism type 3
DHICA	5,6-dihydroxyindole-2-carboxylic acid
IQCA	indole-5,6-quinone-2-carboxylic acid
MBTH	3-methyl-2-benzothiazolinone hydrazone
MD	molecular dynamics
RMSD	root-mean square deviation
RMSF	root-mean square fluctuation
Ni-NTA	nickel–nitrilotriacetic acid

## References

[1] Halaban R., Moellmann G. (1990). Murine and human b locus pigmentation genes encode a glycoprotein (gp75) with catalase activity. Proc. Natl. Acad. Sci. USA.

[2] Sarangarajan R., Boissy R.E. (2001). Tyrp1 and oculocutaneous albinism type 3. Pigment Cell Res..

[3] Boissy R.E., Sakai C., Zhao H., Kobayashi T., Hearing V.J. (1998). Human tyrosinase related protein-1 (TRP-1) does not function as a DHICA oxidase activity in contrast to murine TRP-1. Exp. Dermatol..

[4] Olivares C., Jimenez-Cervantes C., Lozano J.A., Solano F., Garcia-Borron J.C. (2001). The 5,6-dihydroxyindole-2-carboxylic acid (DHICA) oxidase activity of human tyrosinase. Biochem. J..

[5] Lai X., Wichers H.J., Soler-Lopez M., Dijkstra B.W. (2017). Structure of Human Tyrosinase Related Protein 1 Reveals a Binuclear Zinc Active Site Important for Melanogenesis. Angew. Chem. Int. Ed. Engl..

[6] Fang D., Tsuji Y., Setaluri V. (2002). Selective down-regulation of tyrosinase family gene TYRP1 by inhibition of the activity of melanocyte transcription factor, MITF. Nucleic Acids Res..

[7] Patel M.H., Dolinska M.B., Sergeev Y.V. (2021). Tyrp1 Mutant Variants Associated with OCA3: Computational Characterization of Protein Stability and Ligand Binding. Int. J. Mol. Sci..

[8] Wiriyasermkul P., Moriyama S., Nagamori S. (2020). Membrane transport proteins in melanosomes: Regulation of ions for pigmentation. Biochim. Biophys. Acta Biomembr..

[9] Simeonov D.R., Wang X., Wang C., Sergeev Y., Dolinska M., Bower M., Fischer R., Winer D., Dubrovsky G., Balog J.Z. (2013). DNA variations in oculocutaneous albinism: An updated mutation list and current outstanding issues in molecular diagnostics. Hum. Mutat..

[10] Dolinska M.B., Anderson D.E., Sergeev Y.V. (2022). In vitro characterization of the intramelanosomal domain of human recombinant TYRP1 and its oculocutaneous albinism type 3-related mutant variants. Protein Sci..

[11] Dolinska M.B., Wingfield P.T., Young K.L., Sergeev Y.V. (2019). The TYRP1-mediated protection of human tyrosinase activity does not involve stable interactions of tyrosinase domains. Pigment Cell Melanoma Res..

[12] Martinez J.H., Solano F., Garcia-Borron J.C., Iborra J.L., Lozano J.A. (1985). The involvement of histidine at the active site of Harding-Passey mouse melanoma tyrosinase. Biochem. Int..

[13] Kobayashi T., Urabe K., Winder A., Jimenez-Cervantes C., Imokawa G., Brewington T., Solano F., Garcia-Borron J.C., Hearing V.J. (1994). Tyrosinase related protein 1 (TRP1) functions as a DHICA oxidase in melanin biosynthesis. EMBO J..

[14] Schallreuter K.U., Kothari S., Chavan B., Spencer J.D. (2008). Regulation of melanogenesis--controversies and new concepts. Exp. Dermatol..

[15] Smith D.R., Spaulding D.T., Glenn H.M., Fuller B.B. (2004). The relationship between Na(+)/H(+) exchanger expression and tyrosinase activity in human melanocytes. Exp. Cell Res..

[16] Negroiu G., Branza-Nichita N., Petrescu A.J., Dwek R.A., Petrescu S.M. (1999). Protein specific N-glycosylation of tyrosinase and tyrosinase-related protein-1 in B16 mouse melanoma cells. Biochem. J..

[17] Lai X., Wichers H.J., Soler-Lopez M., Dijkstra B.W. (2020). Phenylthiourea Binding to Human Tyrosinase-Related Protein 1. Int. J. Mol. Sci..

[18] Dolinska M.B., Young K.L., Kassouf C., Dimitriadis E.K., Wingfield P.T., Sergeev Y.V. (2020). Protein Stability and Functional Characterization of Intra-Melanosomal Domain of Human Recombinant Tyrosinase-Related Protein 1. Int. J. Mol. Sci..

[19] Ghanem G., Fabrice J. (2011). Tyrosinase related protein 1 (TYRP1/gp75) in human cutaneous melanoma. Mol. Oncol..

[20] Gautron A., Migault M., Bachelot L., Corre S., Galibert M.D., Gilot D. (2021). Human TYRP1: Two functions for a single gene?. Pigment Cell Melanoma Res..

[21] Toyofuku K., Wada I., Valencia J.C., Kushimoto T., Ferrans V.J., Hearing V.J. (2001). Oculocutaneous albinism types 1 and 3 are ER retention diseases: Mutation of tyrosinase or Tyrp1 can affect the processing of both mutant and wild-type proteins. FASEB J..

[22] Varghese P.K., Abu-Asab M., Dimitriadis E.K., Dolinska M.B., Morcos G.P., Sergeev Y.V. (2021). Tyrosinase Nanoparticles: Understanding the Melanogenesis Pathway by Isolating the Products of Tyrosinase Enzymatic Reaction. Int. J. Mol. Sci..

[23] Osuna I., Dolinska M.B., Sergeev Y.V. (2022). In Vitro Reconstitution of the Melanin Pathway’s Catalytic Activities Using Tyrosinase Nanoparticles. Int. J. Mol. Sci..

[24] Woods T., Sergeev Y.V. (2023). Evaluating the Cysteine-Rich and Catalytic Subdomains of Human Tyrosinase and OCA1-Related Mutants Using 1 mus Molecular Dynamics Simulation. Int. J. Mol. Sci..

[25] McCafferty C.L., Sergeev Y.V. (2016). In silico Mapping of Protein Unfolding Mutations for Inherited Disease. Sci. Rep..

[26] McCafferty C.L., Sergeev Y.V. (2016). Dataset of eye disease-related proteins analyzed using the unfolding mutation screen. Sci. Data.

[27] Ortiz F.W., Sergeev Y.V. (2019). Global computational mutagenesis of domain structures associated with inherited eye disease. Sci. Rep..

[28] Krieger E., Darden T., Nabuurs S.B., Finkelstein A., Vriend G. (2004). Making optimal use of empirical energy functions: Force-field parameterization in crystal space. Proteins.

[29] Krieger E., Geretti E., Brandner B., Goger B., Wells T.N., Kungl A.J. (2004). A structural and dynamic model for the interaction of interleukin-8 and glycosaminoglycans: Support from isothermal fluorescence titrations. Proteins.

[30] Humphrey W., Dalke A., Schulten K. (1996). VMD: Visual molecular dynamics. J. Mol. Graph..

