# Supplementary material for: Analysis of Immobilized Protein Unfolding and Molecular Dynamics Shows How pH, Glycosylation, and OCA3-Related Variants Influence Tyrp1’s Stability and Function
Source: Int J Mol Sci. 2026 May 30;27(11):4961. doi: 10.3390/ijms27114961 (PMC13256153; doi:10.3390/ijms27114961)
Supplement: Supplementary file 1 [file ijms-27-04961-s001.zip › ijms-4324283-supplementary.pdf]

Supplementary Information:

Supplementary Table S1. Protein stability of intact Tyrp1 and R356Q at acidic (pH 5) and near-neutral (pH 7) conditions.

**Protein stability changes**	**Acidic conditions**		**Near-neutral conditions**	
	**Tyrp1**	**R356Q**	**Tyrp1**	**R356Q**
**ΔG°, kcal/mol**	1.702	1.281	1.979	1.324
**ΔΔG, kcal/mol**	n/a	0.421	n/a	0.655

Supplementary Table S2. Protein stability of immobilized Tyrp1 and R356Q at acidic versus near-neutral conditions.

Protein stability changes	Acidic conditions		Near-neutral conditions	
	Tyrp1	R356Q	Tyrp1	R356Q
**ΔG°, kcal/mol**	1.444	0.749	2.562	0.564
**ΔΔG, kcal/mol**	n/a	0.695	n/a	1.998

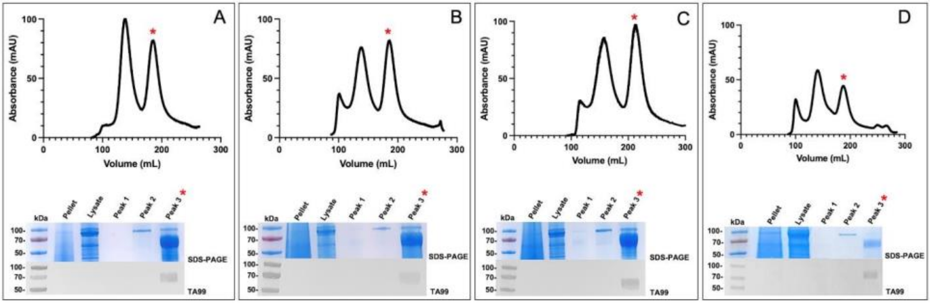

Supplementary Figure S1. Purification profiles of Tyrp1 and mutant lysates. The following fractions were pooled and concentrated: (A) Tyrp1 fractions (175-238 mL), (B) R326H fractions (172-230 mL), (C) D308N fractions (196-260 mL), and (D) R356Q fractions (172-242 mL). The desired protein of interest was identified in the last peak for all samples and noted by the red asterisk. The inserts show the SDS-PAGE (top) and Western blot (bottom) analyses of the pellet, lysate, and three peaks post-purification.

Protein purification: buffer exchange was achieved through the size-exclusion chromatography step using a (HiPrep 26/60 Sephacryl S-300 column, equilibrated in gel filtration buffer: 50 mM Tris-HCl, pH 7.4 or pH 5.0, 150 mM NaCl, 1 mM EDTA, 50 μM TCEP). Pooled SEC fractions were concentrated using Amicon Ultra-15 centrifugal filter units at 5,000 rpm, 4 °C, for 30 min per spin, repeated until the final protein concentration exceeded 1 mg/mL

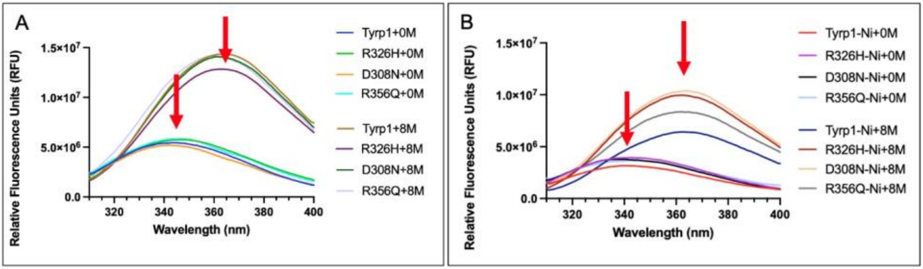

Supplementary Figure S2. Fluorescence shift from 0M to 8M urea. (A) displays intact proteins at 0M and 8M urea concentrations, while (B) shows immobilized proteins under the same conditions. After 24 hours of exposure to urea, both intact and immobilized Tyrp1, R326H, D308N, and R356Q exhibit a fluorescence shift from 345 nm to 365 nm.

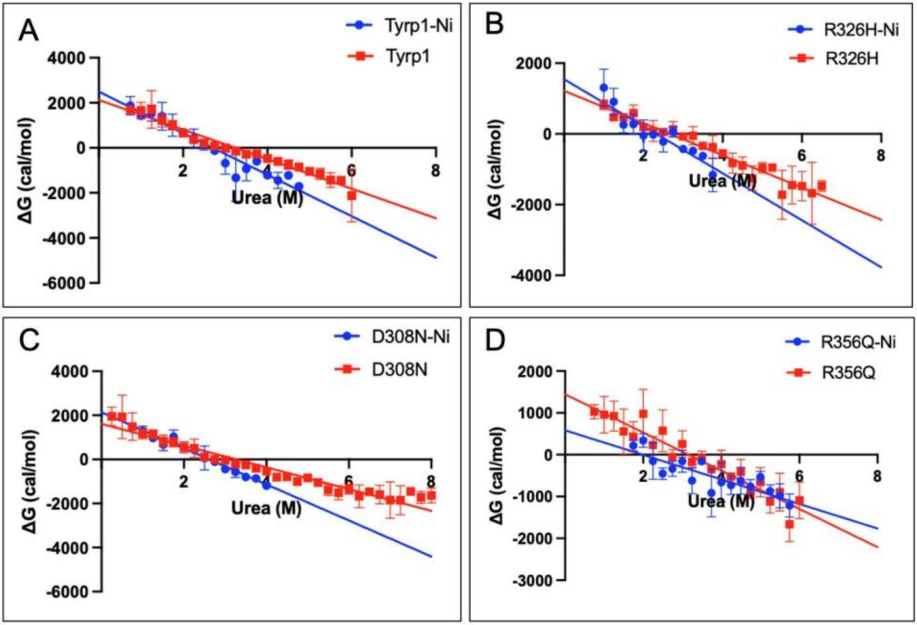

Supplementary Figure S3. Protein stability graphs for immobilized and intact proteins at near-neutral conditions.

Changes in protein stability were obtained as described in the Methods section. Each plot is shown as a function of urea concentration. All data points were measured in triplicate and were averaged, and standard errors were calculated and presented by error bars. Averages are represented by red squares and blue circles. Linear fit to the experimental points shown by solid lines, blue and red, for immobilized and intact proteins, respectively. Panels A-D show how protein stability in water was determined from linear extrapolation at a 0-molar urea concentration.

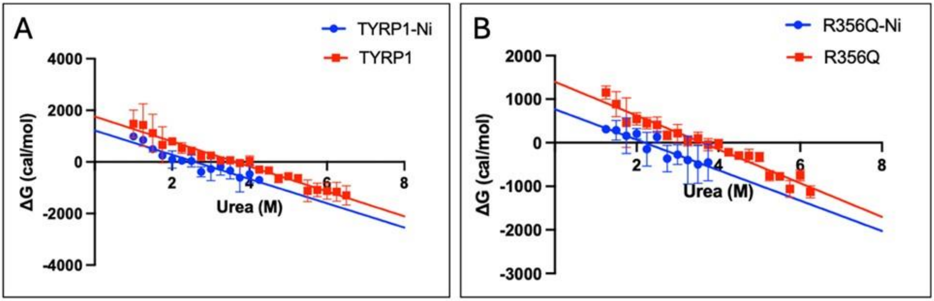

Supplementary Figure S4. Free energy changes (ΔG) from urea-induced unfolding of wild-type Tyrp1 and the R356Q mutant at acidic conditions, measured in the presence and absence of Ni-NTA bead immobilization. (A) Wild-type Tyrp1 immobilized on beads (blue circles) and intact protein in solution (red squares); (B) R356Q mutant immobilized on beads (blue circles) and intact mutant in solution (red squares). ΔG values were determined from linear fits of the unfolding transition regions. Error bars represent one standard deviation.

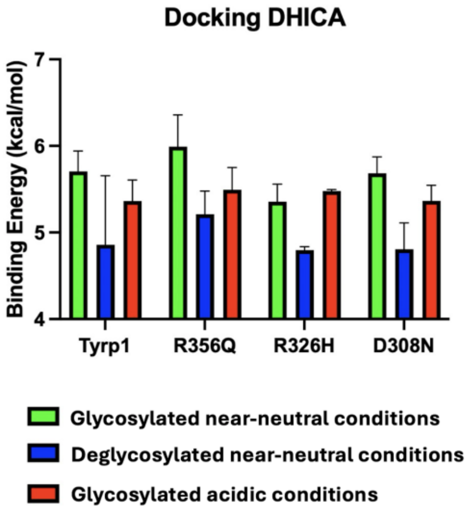

Supplementary Figure S5. Binding energies of DHICA docked to Tyrp1 and its mutants using YASARA (AutoDockLS, 250 runs). Conditions include glycosylated near-neutral (green), deglycosylated near-neutral (blue), and glycosylated acidic (red). Error bars show standard deviation.

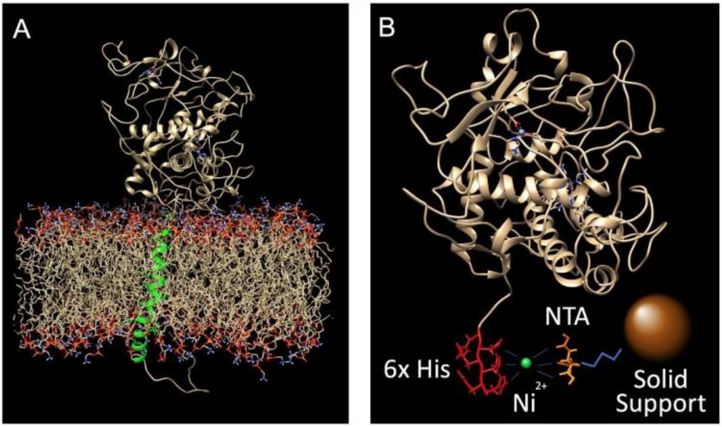

Supplementary Figure S6. Molecular representations of Tyrp1 and its interaction with Ni-NTA particles. (A) displays the full-length Tyrp1 with its transmembrane helix embedded into the membrane, like that observed in the melanosome. (B) illustrates the human recombinant Tyrp1 intra-melanosomal domain immobilized to Ni-NTA particles via the 6xHis tag.
